# Varicella Retinal Vasculopathy: Unilateral Cilioretinal Artery Occlusion Despite Acyclovir Therapy Caught Using Optical Coherence Tomography-Angiography (OCTA)

**DOI:** 10.1155/2019/5752180

**Published:** 2019-07-17

**Authors:** Anadi Khatri, Satish Timalsena, Sudhir Gautam, Muna Kharel

**Affiliations:** ^1^Birat Eye Hospital, Biratnagar, Nepal; ^2^Nepalese Army Institute of Health Sciences, Kathmandu, Nepal

## Abstract

Varicella zoster is known to be associated with vaso-occlusive pathologies, vasculitis, or optic neuritis, leading to profound visual loss. We report a case where a 13-year-old boy who initially presented to us with on and off diminution of vision in his right eye since 3 days and had normal ocular and OCT angiography findings followed up in 5 days with sudden painless diminution of vision in the same eye since one day this time revealing a pale macular region with rest of the retina being normal. Repeated OCT angiography showed loss of the capillary network around the perifoveal region suggesting cilioretinal artery occlusion.

## 1. Introduction

Varicella-zoster virus (VZV) is an exclusively human virus that belongs to the *α*-herpes virus family [1]. VZV is present worldwide and is highly infectious. The primary infection leads to acute varicella or “chickenpox”, usually from exposure either through direct contact with a skin lesion or through airborne spread from respiratory droplets [2].

Ocular manifestations of the pathology have been reported in forms of retinal vasculopathy, retinitis, and optic neuritis [3–7]. Cilioretinal artery occlusion in the pediatric population is considered very rare and it is most often associated with hypercoagulable states and embolic phenomena [8]. We present a patient with profound unilateral vision loss due to cilioretinal artery occlusion following varicella zoster infection.

## 2. The Case

A thirteen-year-old boy presented to us with on and off diminution of vision in the right eye (RE) for 3 days. He had recently had varicella zoster (chicken pox) 7 days back for which he had visited pediatrician and dermatologist. He was on a dose of 400 mg twice daily at the time of presentation but gives history of taking 400 mg for five times initially for 5 days which was appropriate for his body weight of 23 kilograms.

On general evaluation, he had resolving scab marks following varicella zoster dermatitis over the skin of forehead and trunk region. He denies any history of ocular /facial/oral trauma, lightheadedness, or blackouts. The patient party denies observing any abnormal bodily movements or loss of consciousness. The patient does give a history of fever 6 days back which resolved after taking paracetamol. There is no other significant medical or surgical history. Ocular examination revealed a visual acuity of 6/6 in both the eyes (OU). Anterior and posterior segment findings were normal.

**Figure 1:**
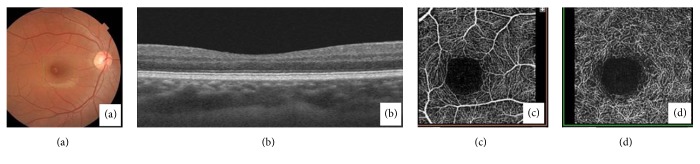
(a)Fundus photo and OCT of macula (b) at initial presentation. Note the well-described perifoveolar capillary plexus in both superficial and deep plexus (c and d).

**Figure 2:**
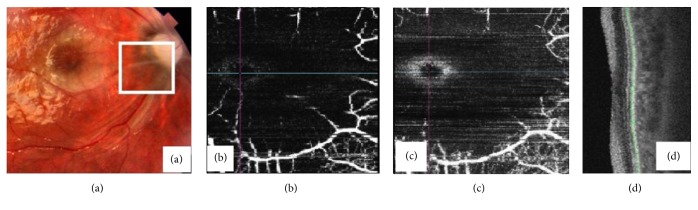
(a) “Ghost-” like cilioretinal vessel(Boxed) impression secondary to occlusion. Note the loss of the perifoveolar capillary plexuses in both superficial and deep plexus (b)and (c) with edema of the perimacular nerve fibre layer(d).

Dilated fundus examination revealed normal findings (Figure 1(a)). The patient was advised for OCT angiography (Topcon Medical Systems -Triton™ DRI PLUS SS-OCT) which was normal (Figures 1(b), 1(c), and 1(d)). He was advised for blood examinations including total blood count, peripheral blood smear, CRP, homocysteine levels, lipid profile, and cardiological evaluation. The patient followed up days later with sudden painless diminution of vision in RE. He had a vision of 1/60 RE and 6/6 in the left eye (LE). The anterior segment showed a relative afferent pupillary defect in RE (Grade 3, (Bell's classification)). There was no evidence of vitritis. Dilated fundus examination of RE revealed retinal edema involving the posterior pole with distinctive cherry red-like spot at the foveolar area. The disc was slightly pale. There were arteriolar attenuation and segmentation of blood column within the arterioles (Figure 2(a)). The LE was normal. His blood parameters and cardiovascular and serological reports were normal.

Homocysteine levels and coagulation profile were in the normal range. We planned for FFA but the patient and his party denied going under the procedure and hence we advised for repeating the OCT angiography. OCTA revealed loss of capillary plexus in both superficial and deep retinal layers (Figures 2(b) and 2(c)). Optical coherence tomography revealed massive retinal thickening due to edema with disorganized retinal elements (Figure 2(d)). A diagnosis of right eye cilioretinal artery occlusion was made on the basis of clinical and OCTA findings. Despite treatment with acyclovir and steroids, the vision failed to improve until 4 weeks after which the patient was lost to follow-up.

## 3. Discussion

Retinal artery occlusions (RAO) are most commonly a result of embolic obstruction. Other mechanisms are also known and mainly include exogenous emboli, thrombotic, vasospastic, and vasculitic pathologies [9]. Retinal artery occlusion has been reported as a complication of varicella-zoster infection in various age groups denoting vaso-occlusive nature of pathology [3–5, 7, 10]. It is also one of the components of the varicella vasculopathy [5].

RAO and branched-RAO have been reported in the pediatric population following chickenpox infection. Jayaram H. et al. have reported bilateral ophthalmic artery occlusion secondary to a probable chicken pox vasculopathy in a child [5]. Similarly, Zamora et al. [3] have reported a case where the patient had multiple recurrent branched retinal artery occlusion secondary to varicella zoster infection. Sebban AI et al. [4] have also reported a similar case with a branch retinal artery following varicella zoster eruptions. Lalit et al. [7] from our region have also reported a case of CRAO with a profound vision loss in a 12-year-old patient who presented within few days after varicella zoster infection with dermatitis causing a profound vision loss.

Our patient presented with unilateral cilioretinal artery occlusion. This was diagnosed with the clinical finding of the fundus and confirmed by OCTA. This is the first case of this pathology to be reported with the use of OCTA. Cilioretinal artery occlusion is considered as the least common variant of RAO [11]. In addition to this rarity, the patient in the first visit had complained about on and off diminution of vision in the same eye for which we performed OCTA which revealed completely normal retinal vasculature architecture. The systemic evaluation had also revealed normal findings. As the patient was already on antiherpetic agents, a continuation of the same medication was advised. Disregard of this, the patient followed up in just a few days with RAO. OCTA showed loss of capillary plexuses in both superficial and deep layer analysis. The presentation of our patient is congruent in many terms with varicella vasculopathy. It has been mentioned that varicella vasculopathy may present as transient ischemic attack with neurological and retinal deficits [10, 12]. A closely related term, postvaricella angiopathy, has been also described in the literature where vaso-occlusive pathologies can occur after several months following chicken pox [10].

Most literatures [2, 3, 7, 10, 13, 14] agree that retinal complications can be treated or prevented with the use of Acyclovir. The patient was already under treatment of this drug and was advised to continue the medication. Yet, the patient presented with cilioretinal artery occlusion in one eye. Studies have suggested that postvaricella vasculopathy is associated with stenosis of the cerebral arteries and also account for nearly 1/3rd of childhood strokes [10, 12, 13]. They have also concluded that in such cases antithrombotic therapy may prevent strokes/transient ischemic attacks (TIA) [13]. The same may hold true for retinal vasculature and a detailed study of retinal vasculatures using fluorescence angiography or OCTA modules may be necessary to confirm this.

## 4. Conclusion

Varicella zoster infections are an established cause for retinal artery occlusions in the pediatric population and can also cause cilioretinal artery occlusion. Use of acyclovir alone may not suffice to prevent vaso-occlusive phenomena and addition of antithrombotic may also be needed. OCTA can be a good noninvasive tool to aid in diagnosis.
